# Pregnancies, abortions, and pregnancy intentions: a protocol for modeling and reporting global, regional and country estimates

**DOI:** 10.1186/s12978-019-0682-0

**Published:** 2019-03-20

**Authors:** Jonathan Marc Bearak, Anna Popinchalk, Gilda Sedgh, Bela Ganatra, Ann-Beth Moller, Özge Tunçalp, Leontine Alkema

**Affiliations:** 1 Guttmacher Institute, 125 Maiden Ln, New York, NY 10038 USA; 2 Department of Reproductive Health and Research, World Health Organization, Avenue Appia, 20 Geneva, Switzerland; 3 Department of Biostatistics and Epidemiology, University of Massachusetts–Amherst, Amherst, MA 01003 USA

**Keywords:** Pregnancy, Abortion, Family planning, Unintended pregnancy, Bayesian, Estimates, Trends, National, Regional, Global

## Abstract

**Background:**

Estimates of pregnancies, abortions and pregnancy intentions can help assess how effectively women and couples are able to fulfil their childbearing aspirations. Abortion incidence estimates are also a necessary foundation for research on the safety of abortions performed and the consequences of unsafe abortion. Furthermore, periodic estimates of these indicators are needed to help inform policy and programmes.

**Methods:**

We will develop a Bayesian hierarchical times series model which estimates levels and trends in pregnancy rates, abortion rates, and percentages of pregnancies and births unintended for each five-year period between 1990 and 2019. The model will be informed by data on abortion incidence and the percentage of births or pregnancies that were unintended. We will develop a data classification process to be applied to all available data. Model-based estimates and associated uncertainty will take account of data sparsity and quality. Our proposed approach will advance previous work in two key ways. First, we will estimate pregnancy and abortion rates simultaneously, and model the propensity to abort an unintended pregnancy, as opposed to modeling abortion rates directly as in prior work. Secondly, we will produce estimates that are reproducible at the country level by publishing the data inputs, data classification processes and source code.

**Discussion:**

This protocol will form the basis for updated global, regional and national estimates of intended and unintended pregnancy rates, abortion rates, and the percent of unintended pregnancies ending in abortion, from 1990 to 2019.

## Plain English summary

This protocol describes how we propose to estimate global levels and trends in the incidence of pregnancy, abortion and intended and unintended births in 1990–2019. Such estimates can help assess how effectively women and couples are able to fulfil their childbearing aspirations. Abortion incidence estimates are also a necessary foundation for research on the safety of abortions performed and the consequences of unsafe abortion. Estimates can additionally inform policy and programmes, such as by highlighting the importance of access to safe, legal abortion care, a critical reproductive health service.

Estimating the distribution of pregnancies by intention and outcome is challenging. Data requirements include information on the proportion of births that are intended and on the incidence of abortion. Countries may lack data on one or both of these variables, for some or all time periods in question [1, 2]. Additionally, documenting the reliability of abortion statistics can be challenging.

Rigorous methodologies are needed for the estimation of these imperfectly measured outcomes. We will develop a statistical model that will be informed by data on abortion incidence and the percentage of births or pregnancies that were unintended. We will develop a data classification process to be applied to all available data. Model-based estimates and the ranges around the estimates will take account of data sparsity and quality. Our proposed approach will advance previous work in two key ways. First, we will estimate unplanned birth and abortion rates simultaneously, as opposed to modeling abortion rates directly, and using those estimates as a basis for estimating unplanned birth rates, as in prior work. Secondly, we will produce estimates that are reproducible by publishing the data inputs, data classification processes and source code.

Improving upon previous work [1, 2], this protocol will form the basis for transparent and replicable global, regional and national estimates of intended and unintended pregnancy rates, abortion rates, and the percent of unintended pregnancies ending in abortion from 1990 to 2019.

## Background

### Background, rationale, aims and objectives

Estimates of pregnancies, abortions and pregnancy intentions can help assess how effectively women and couples are able to fulfil their childbearing aspirations. Abortion incidence estimates are also a necessary foundation for research on the safety of abortions performed and the consequences of unsafe abortion. Furthermore, periodic estimates of pregnancies, abortions and pregnancy intentions are needed to help inform policy and programmes.

However, estimating the distribution of pregnancies by intention and outcome is challenging. Data requirements include information on the proportion of births that are intended and on the incidence of abortion. Countries may lack data on one or both of these variables, for some or all time periods in question [1, 2]. Additionally, documenting the reliability of abortion statistics can be challenging [1]. Regional and sub-regional estimates of abortion incidence and unintended pregnancies were published without any country estimates in 2016 and 2018, respectively [1, 2].

Our new approach allows us to incorporate evidence on the incidence pregnancies and abortions, as well as pregnancy intentions, so that our estimates for each of these related indicators are informed by the available data on all indicators. Our model-based approach makes it possible to produce country estimates along with the certainty of these estimates. This in turn should increase the utility of the findings to policymakers, researchers and other stakeholders. We additionally describe a new process for classifying abortion data which allows us to extract additional details for use in the model.

The Sustainable Development Goals call for universal access to sexual and reproductive healthcare services as a priority, including reducing unmet need for contraception [3]. Our planned estimates can provide additional insights related to this target. Moreover, access to safe, legal abortion is a critical reproductive healthcare service. Our estimates can highlight where more resources are needed. This manuscript presents the protocol which will be used to produce estimates.

## Text box: Definitions

**Table:** 

*Pregnancies:* Pregnancies are comprised of live births, abortions and miscarriages. Abortions refer to those that are induced, while miscarriages refer to spontaneous fetal losses after 5 weeks of gestation, including stillbirths.	
*Unmet need*: Women who want to stop or delay childbearing but are not using any method of contraception are defined as having an unmet need for contraception.	
*Intended pregnancy*: We classify a pregnancy as intended if a woman reports that at or just before the time of conception, she wanted to become pregnant.	
*Unintended pregnancy*: The remainder of pregnancies are classified as unintended. Theoretically, these roughly correspond to the pregnancies which occur to women who are using or who have an unmet need for contraception. However, pregnancy intentions can be fluid and fall along a spectrum, such that the available family planning indicators may not perfectly align with measures of pregnancy intentions.	
*Unintended births*: We refer to live births that follow unintended pregnancies as unintended births.	
*Marriage:* Married women include those living in a cohabiting union. This is consistent with the definition employed by the DHS and by the UNPD [19, 20].	

## Method

### Data sources

Multiple data sources will be employed for this analysis. Abortion data may be obtained from published studies or official statistics [1]. Official statistics are obtained from Ministries of Health and National Statistical Offices [4]. When official statistics cannot be found or are not easily accessible, questionnaires are sent to country contacts at Ministries of Health, National Statistical Offices or Reproductive Health experts. If not otherwise available, official statistics may sometimes be obtained from the UNSD Demographic Yearbook [5]. Published studies will be obtained by searching PubMed and Google Scholar for the terms “abortion incidence,” “abortion estimates,” “termination of pregnancy,” “induced abortion,” and “menstrual regulation,” followed by, one by one, the name of each country.

Data on the share of births and pregnancies intended or unintended are compiled from surveys done periodically in developing and developed countries, and from one-time studies that are found through a PubMed and Google Scholar literature search [2]. We obtain all publicly available microdata from the Demographic and Health Surveys (DHS) as well as the Multiple Indicator Cluster Surveys (MICS). Where the surveys are restricted, we will also obtain data from Reproductive Health Surveys (RHS) and DHS reports.

Estimates of the number of women of reproductive age, the percent of these women who are married, and the percent of married women with unmet need for contraception, no contraceptive need, and met need, by country and year, for women aged 15–49, as well as the numbers of live births, are provided by the UNPD [6–8].

### Modeling strategy

**Fig. 1:**
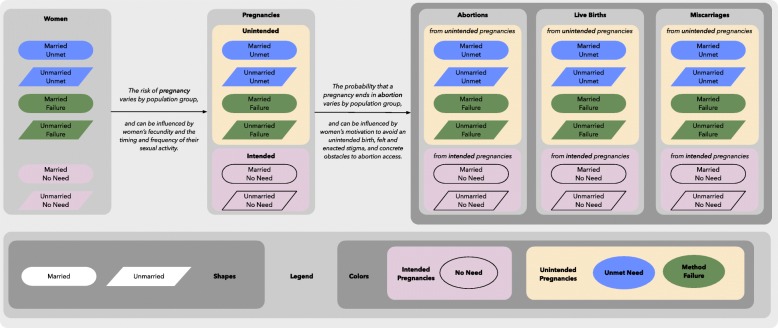
Theoretical framework

Our model is grounded in a theoretical framework in which the incidence of unintended pregnancy is a function of the numbers of women with an unmet need for contraception and women using a contraceptive method who experience a method or user failure, separately by marital status, and the risk of pregnancy in each of these population groups *(see* Fig. 1*)*. Similarly, the incidence of intended pregnancy is a function of the number of women with no need for contraception, separately by marital status, and their risk of pregnancy*.*

Thus, the number of pregnancies Ω to occur in country *c* during five-year time period *t* is equal to the sum of pregnancies across all population groups. Algebraically*,* where Ω^*f*^ is the number of pregnancies to occur in population group $f,{\Omega}_{ct}={\Sigma}_f\kern0.1em {\Omega}_{ct}^f$

The number of pregnancies to occur in a population group is in turn a function of the number of women in that group, *w*_*fct*_, and their risk of pregnancy, *ω*_*fct*_:

$$ {\Omega}_{ct}^f={w}_{fct}\ {\omega}_{fct}. $$

The incidence of abortion within a population group, Ψ^*f*^, is a function of the numbers of pregnancies in that group and the group-specific probability that a pregnancy will end in an abortion, α_*f*_:

$$ {\Psi}_{ct}^f={\Omega}_{ct}^f\ {\upalpha}_{fct}. $$

The incidence of abortion in a country-period is in turn the sum of the numbers of abortions across population groups, ${\Psi}_{ct}={\Sigma}_f{\Psi}_{ct}^f$. Alternatively, replacing Ψ^*f*^with the above equations, the incidence of abortion can be expressed as the summation across all population groups of the product of the number of women, the risk of pregnancy, and the probability that a pregnancy ends in abortion,

$$ {\Psi}_{ct}=\sum \limits_f{w}_{fct}\ {\omega}_{fct}\ {\upalpha}_{fct}. $$

Pregnancy outcomes are given by abortions, live births, or miscarriages. In our model framework, live births (Fig. 1, 4th column) are given by UNPD estimates [9]. Consistent with previous pregnancy estimates [2, 10], we estimate miscarriages using an approach derived from life tables of pregnancy loss by gestational age in which there is, on average, one miscarriage for every ten abortions, and one for every five live births [11–13].

Marital status, contraceptive need and use, and abortion are key proximate determinants of pregnancy rates and fertility [14]. However, the sizes of these population groups will not explain all differences between time periods or between countries. The risk of pregnancy in these population groups can be influenced by women’s fecundity and the timing and frequency of their sexual activity [14]. Additionally, the percent of unintended pregnancies which end in abortion may vary according to differences in women’s motivation to avoid an unintended birth, social and personal stigma, and concrete obstacles to abortion access. [15] Therefore, we will consider covariates which may proxy these factors. Candidate covariates include:

- Gross domestic product per capita
- Human Development Index
- Female literacy rate
- Gender inequality index
- Urban population
- Legal abortion status

Available covariates are unlikely to be able to explain all variability across countries and within countries over time in pregnancy rates and probabilities of aborting an unintended pregnancy for two main reasons. First, information on determinants is limited, i.e. available covariates will be proxy covariates at best. Second, covariates may be estimated imperfectly and are subject to uncertainty. As a result, there will be unexplained heterogeneity across countries and within countries over time.

We will address the issue of unexplained heterogeneity in our outcomes—subgroup estimates of pregnancy rates and propensities to abort— with a Bayesian hierarchical time series model. After accounting for covariates, we expect temporal correlations in the unexplained fluctuations. This will be captured through a time series model on subgroup outcomes. Similarly, we expect similarities across countries within subregions in the unexplained fluctuations. We will use a hierarchical model to estimate country parameters, such that information is exchanged across countries within the same group. Countries in which the statistical relationships are expected to be similar will be grouped together, and these may differ from geographic subregions.

We will use a Bayesian framework to (i) implement the modeling strategy for the unknown outcomes as explained above, and (ii) incorporate all available data, as well as the uncertainty associated with each datum. Estimates for pregnancies will be consistent with information on pregnancy outcomes, i.e. the total of abortions, live births, and associated miscarriages. The model will include data on abortion incidence, the percent of live births that were intended, and data on the distribution of outcomes by population group to calibrate the group-specific rates. The Bayesian approach will produce point estimates that combine information directly from data for the respective country-period with information from other periods and countries. Uncertainty intervals around each of our estimates account for the quantity and quality of all available data, as well as the unexplained heterogeneity across countries and periods.

#### Model validation and selection of covariates

We will assess model performance using a combination of validation exercises and visual inspection of plots. Validation exercises will include a comparison of the model-based estimates produced using the complete dataset to estimates produced by excluding random subsets of the data. Additionally, we will compare the model-based estimates for each country to the estimates produced by excluding a country’s data. The goal of these comparisons is to assess whether the model-based estimates are unbiased and whether the model produces an appropriate uncertainty assessment for countries and periods where data are unavailable. Criteria for inclusion of covariates will include the minimization of error and bias, as well as their theoretical rationale and other considerations.

### Classification of abortion data

**Fig. 2:**
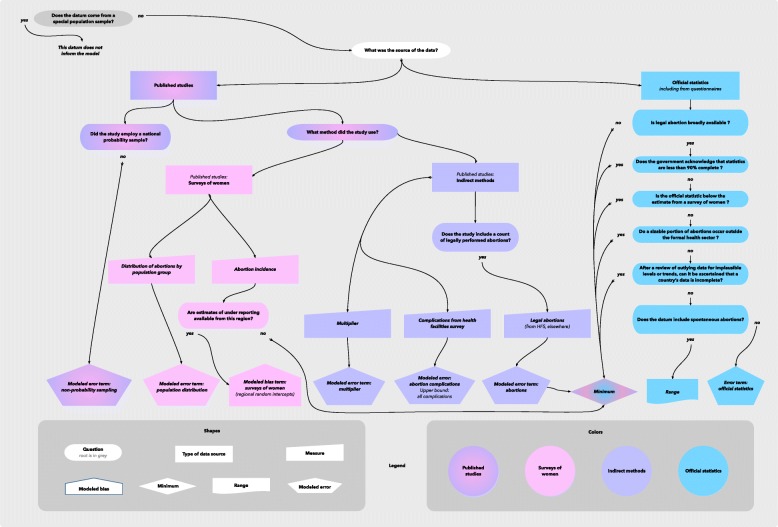
Classification of abortion data

The reliability of abortion data varies widely so that each datum must be classified to determine how it informs the estimates in our statistical model, and we developed a logic to address this issue. We first address, “Does the datum come from a special population sample?” (e.g., a high-risk population) *(see* Fig. 2*).* If so, the datum does not inform the model. If not, we then ask, “What was the source of the data?” The diagram includes additional sequences which address issues unique to published studies and official statistics, respectively.

(1) *Published Studies:* If the datum comes from a published study, we first ask, “Did the study employ a national probability sample?” If the study did not employ a national probability sample, we include an additional error term. This allows, for example, estimates from a subnational study or a national non-probability sample to inform our model, but less so than a national probability study would. Our model can thus weight other information more heavily relative to such a survey when calculating country estimates, and, as is further appropriate, produce wider uncertainty intervals for a country with lower-quality data.

Our decision logic includes sequences to address additional issues which apply regardless of whether the study used a national probability sample. The sequences differ depending on whether the study estimated abortion rates from women’s reports or used an indirect approach.

*(1a) Published studies which use women’s reports:* The pink sequence addresses issues applicable to a datum which comes from a survey of women. These studies may report, in addition to overall abortion rates, information on the distribution of abortions by subgroup; we include an additional error term for any such datum, so that the model acknowledges that abortion under-reporting may vary by subgroup [16]. For an estimate of the overall rate, our methodology considers whether it is possible to estimate an inflation factor whose expectation is equal to the average level of under-reporting in a survey. The appropriate inflation factor may vary across surveys; for example, abortion stigma may be much higher, on average, in a region where abortions are more stigmatized. If no estimates to inform the inflation factor are available for this datum’s modelling region, then, the datum provides our model with a minimum estimate of abortion incidence. If estimates of under-reporting *are* available in the region – because of the coincidence of a survey of women with a reliable official statistic – then, this information is included as a prior, with information on under-reporting exchanged hierarchically within regions. Regional inflation factors are determined within the model in order to estimate the additional uncertainty associated with inflation.

*(1b) Indirect methods:* The lavender branch describes how we incorporate estimates from indirect methods. Most extant indirect studies use the abortion incidence complications method (AICM) [17], and this branch of our decision tree specifically describes the decisions relevant to the AICM. This method was originally developed to estimate abortion incidence in countries where abortion is highly restricted. Data is collected on the number of women treated in health facilities for abortion complications in a given period. Additionally, information from a survey of health professionals is used to estimate the proportion of women obtaining abortions who have complications and who obtain treatment at a medical facility. The inverse of this statistic is the estimated ratio of the number of abortions to the number of abortions which resulted in complications treated in a health facility. An abortion rate is estimated by multiplying this ratio with the estimated number of women treated in facilities.

The uncertainty attributable to the multiplier is unknown and is not incorporated into the published estimates from these studies. For the purpose of our model, as the percent of abortions which result in treated complications differs across studies, so should the uncertainty in the estimated abortion rate. For this reason, we include distinct error terms for the multiplier and for the number of complications, rather than entering the published estimate directly into our model, wherever possible.

The error term for the number of complications is asymmetric, truncated on the right. Some of the complications recorded in the health facilities may follow from miscarriages. The study’s authors subtract these before estimating the abortion rate. Some of the uncertainty may be associated with this adjustment, as this adjustment is contingent upon assumptions. As such, the upper bound of the error for the number of complications is the total number of complications (i.e., including those that result from miscarriages).

In countries where abortion is broadly legal but there are still high levels of unsafe abortion, a modified AICM is employed which incorporates information on the number of legal abortions. We will include an additional error term to allow for uncertainty in the measurement of the number of legal abortions, and we treat such a datum as a minimum estimate of the overall abortion rate. The implication of this is that the uncertainty in the estimated abortion rate is larger above the point estimate than below.

(2) *Official statistics:* We would like to treat all abortion data as point estimates, but due to various issues including legality and underreporting, official statistics may not include all abortions. Such data are classified as minimum estimates; these inform the model that the true abortion rate is no less than the observed rate. Our data classification process for official statistics, drawn in light blue, describes how each datum is classified as either a point or minimum estimate of abortion incidence. Table 1 lists each question from the diagram and describes how these are handled.

**Table 1:** Data classification process for official statistics on abortion incidence

Question from Flowchart	Process
Is legal abortion broadly available?	If legal abortion is not broadly available, the datum is classified as a minimum estimate of abortion incidence. If legal abortion *is* broadly available, then it is possible that the data are complete, and we proceed to the next question.
Does the official report acknowledge that statistics are less than 90% complete?	If the government acknowledges that an abortion datum is incomplete, counting fewer than 90% of abortions, in their official report or in their response to the questionnaire we distribute to its statistics office, then the datum is classified as a minimum. If the government claims that its statistics *are* complete, then, we proceed to the next question.
Is the official statistic below the estimate from a survey of women?	If the officially reported number of abortions is smaller than the number of abortions estimated in a published study based on women’s reports, then, all years of official statistics from that country are coded as minimum estimates of abortion incidence unless it can be determined that the quality of official statistics was poor in a specific period. If the official report exceeds the number estimated from women’s reports or such a study is unavailable, then we proceed to the next question.
Do a sizable portion of abortions occur outside the formal health sector?	A datum may be classified as a minimum estimate in light of evidence that a sizable portion of abortions occur outside the formal health sector. For example, even if abortions are legally available in the public sector, medical abortions may occur in the private sector that are not counted in official statistics. If not, we proceed to the next question.
After a review of outlying data for implausible levels or trends, can it be ascertained that a country’s data is incomplete?	Outlying data will be reviewed by the study team and the technical advisory group. If information on levels or trends is ascertained to be implausible, such data will be classified as a minimum estimate of abortion incidence. Otherwise the datum will be treated as a point estimate with an error term.
Does the datum include spontaneous abortions?	If the datum includes spontaneous abortions in addition to induced abortions, then, the model revises this number downward based on the formula, *no. of pregnancies* = 1.2 *no. of live births* + 1.1 *no. of induced abortions*, and this datum is treated as a minimum. This downwardly-revised datum is treated as a minimum based on the expectation that this adjustment subtracts abortions as well as miscarriages. Additionally, the unadjusted statistic is treated as a maximum. I.e., where *b* represents the number of births and *m* represents the number of abortions including miscarriages, the abortion rate lies along the interval [(b - 1.1 *m) ÷ 1.2*, *m]*. Note that this issue may also be relevant to a datum which is classified as a minimum during a previous step in this process; in such cases, the minimum is also adjusted as per this equation.

If new studies should be uncovered that employ approaches (or methodologies) not addressed in this decision logic, we will expand the logic to incorporate these studies and we will employ the principles discussed here to ascertain how to treat data from such studies.

### Classification of pregnancy intention data

**Fig. 3:**
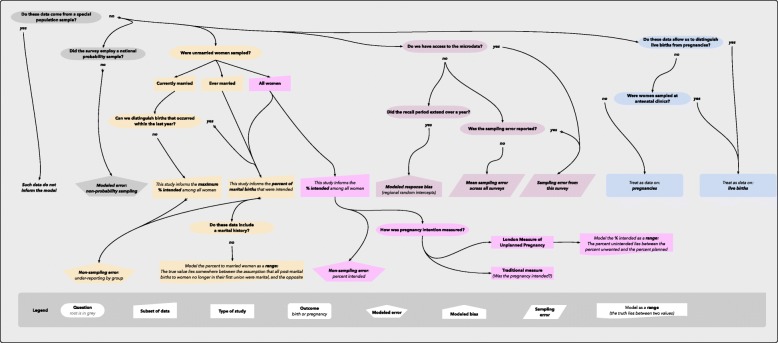
Classification of pregnancy and birth intention data

The decision logic for classifying pregnancy intention data consists of five sequences in grey, yellow, pink, lavender and blue *(see* Fig. 3*)*. The leftmost sequence, in grey, contains questions that are also part of the abortion data classification process. We exclude data from a special population sample. We also include an additional error term if the survey did not employ a national probability sample. Whereas the abortion data decision logic discusses how we handle each datum, the pregnancy intention decision logic discusses how we handle data – we make this distinction because we obtain information on pregnancy intention by directly processing microdata wherever possible.

As discussed earlier, our model combines data on abortion incidence, the number of live births, and the percent of live births that were intended at the time a woman became pregnant. The decision logic for the classification of pregnancy intention data refers to the latter: we analyze datasets in which the unit of analysis is, in general, live births that occurred within a year of the interview. Data on live births is preferable to data on pregnancies since, in surveys of women, abortion under-reporting may downwardly bias the estimated percent of pregnancies unintended. However, we may not always have access to microdata. Where necessary, our model will be able to accept data on the percent of pregnancies, rather than live births, (un)intended. This is clarified in the rightmost sequence, shaded in blue, where we additionally note that if women are sampled in antenatal clinics (where abortion can be assumed an improbable outcome), it is preferable to treat the published datum as information on the percent of births unintended (as to do otherwise would upwardly bias the percent of pregnancies intended).

The lavender sequence, second from right, begins by asking “Do we have access to the microdata?” If we do, or if the sampling error was reported in a published study, then we can input the survey’s sampling error into the model. If not, we input the mean sampling error across all surveys. This is combined with one or more additional terms for non-sampling error mentioned at other points in the diagram.

If we do not have access to the microdata, we also ask “Did the recall period extend over a year?” As a child ages, this can increase the odds that a woman reports that a pregnancy was wanted at the time of conception [2]. To minimize the potential for response bias, as well as for recall error, wherever possible we analyze data on live births that occurred within the last year. Where we obtain data from published studies or reports, however, and do not have access to the microdata, this could lead to over-estimating the percent intended and limit our ability to make comparisons across countries. For these inputs, therefore, we will additionally estimate the average response bias, and the additional uncertainty associated with having to model this, using a multi-level model with regional random intercepts.

The yellow branch begins by asking, “Were unmarried women sampled?” This question is necessary because some DHS surveys interview women only if they are currently married or include unmarried women only if they have previously been married. We refer to the latter as surveys of “ever married” women.

The yellow branch expands into the pink branch to address surveys in which women are interviewed regardless of their present or past marital status. These surveys inform the model as to the percent of events – live births or pregnancies, depending on the survey – among all women. As is noted in the diagram, we include an error term for non-sampling error. Additionally, we ask, “How was pregnancy intention measured?” The traditional measure employed by most surveys classifies a pregnancy or birth as intended if a woman retrospectively reports that it was wanted at or just before the time of the conception. However, a handful of surveys may employ the London Measure of Unplanned Pregnancy (LMUP) [18]. The LMUP classifies the conception as planned, ambivalent, or unwanted based on the sum of a woman’s responses to a dozen questions. The ambivalent category includes pregnancies that would have been classified as intended, as well as pregnancies that would have been classified as unintended, using the traditional measure. Therefore, these surveys are input into the model as a range rather than as a point estimate: the percent unintended on the traditional measure lies between the between percent unwanted and the percent planned on the LMUP.

The remainder of the decision tree, in yellow, concerns the percent distribution of intended births by marital status. Studies of all women as well as studies of ever married women inform the percent of marital births intended. Surveys of currently married women also inform this statistic, provided that in such a survey, we can distinguish between births that occurred within the past year: this is because these surveys are in countries (in sub-Saharan Africa or the Middle East) in which divorce is extremely unlikely, particularly just after the birth of a child. For surveys of currently married women in which we cannot exclude older births, the study informs the maximum percent intended among all women: this is based on the assumption that in these countries the percent of nonmarital births intended is no higher than the percent of marital births intended.

Many studies will inform us as to the percent of marital births that were intended. These include surveys of women, surveys of ever married women, and some surveys of currently married women. However, a few studies may inform us as to the maximum percent intended among all women. This will be the case the survey interviewed currently married women and we do not have access to their microdata. In all cases we include an error term for non-sampling error to allow for the potential that under-reporting of pregnancy intention may vary for subgroup, to a degree that may not be the same as the non-sampling error for the percent intended across all women.

Finally, if a survey includes data on the percent of marital births that were intended, we ask, “Do these data include a marital history?” Surveys in the low-income and middle-income countries, namely the DHS and the MICS, typically ask the date of each birth, the date of first union, whether a woman is currently married, and whether a woman (married or unmarried) has been previously married. Any birth to a woman not yet married, and any birth that occurred prior to the date of first union, is clearly a nonmarital birth. Similarly, any birth that occurred subsequent to the date of first union, to a woman who remains with her first partner, is clearly a marital birth. However, if a woman, remarried or not, is no longer within her first union, it is not clear whether a birth subsequent to the date of first union is marital or nonmarital. For this reason, where we analyze data which excludes a marital history, we input the percent of births intended among married women as a range rather than as a point estimate. This means that the true value lies somewhere between the assumption that all post-marital births to women no longer in their first union were marital, and the opposite assumption. In practice, we expect this range to be small, particularly in sub-Saharan African countries. This approach is preferable because the empirical basis is clear, in contrast to either ignoring this valuable data, or to making an informative assumption to extrapolate from a women’s marital status at the time of interview to her marital status at the time of births.

## Results

### Presentation of results

For each five-year period, we will present global, regional, and national estimates of pregnancies, live births, abortions and miscarriages *(see* Table 2*)*. We will evaluate the added value of publishing point estimates for countries with limited data availability based on model findings. We will always present our model-based estimates, which will take into account all available information including information on the uncertainty of each datum. These can differ from the estimates which are included as inputs.

### Project management

Guttmacher and WHO will collaboratively lead this project and all coauthors will be substantively engaged in all aspects of the research. Additionally, a technical advisory group comprised of international experts on fertility and abortion will provide oversight and input into the data classification and model development processes.

**Table 2:** List of model-based indicators to be published

Outcome	Rates	Percentages
Pregnancy	Pregnancy rate Unintended pregnancy rate Intended pregnancy rate	Percent of pregnancies that are unintended Percent of pregnancies that are intended
Abortion	Abortion rate	Percent of pregnancies that end in abortion Percent of unintended pregnancies that end in abortion
Birth	Unintended birth rate Intended birth rate	Percent of live births that are unintended Percent of live births that are intended
Miscarriage	Miscarriage rates	Percent of pregnancies that end in miscarriage

## Discussion

Recent studies by Sedgh et al. and Bearak et al. brought model-based inference to the global and regional estimation of abortion and unintended pregnancy [1, 2]. Their approaches allowed them to make formal inference, present uncertainty intervals, and examine the robustness of their results.

Our approach makes several methodological advances. Whereas Sedgh and colleagues modeled abortion rates by population group, in this study, we model the percent of pregnancies ending in abortion by population group. One implication of this is that whereas Sedgh et al. assumed that group-specific abortion rates were more similar among countries within the same subregion, our approach assumes that group-specific *propensities for a pregnancy to end in abortion* are more similar among countries within the same subregion. Like Bearak and colleagues, we model pregnancy rates for population groups, but whereas they treated abortion estimates as known quantities, our approach jointly estimates both indicators.

Our estimates of pregnancies, abortions, and pregnancy intentions can help to monitor progress toward universal access to reproductive healthcare. This includes monitoring progress toward women’s and couple’s ability to achieve their childbearing aspirations. Moreover, abortion incidence estimates are also a necessary foundation for research on the safety of abortions performed and the consequences of unsafe abortion. These estimates help emphasize the importance of access to safe, legal abortion care, a critical reproductive health service. This work also represents substantive methodological and practical advancements, including through full transparency, improved use of data, a statistical model that more closely reflects the underlying demographic processes, and producing national estimates.

## Abbreviations

DHS: Demographic and Health Surveys
LMUP: London Measure of Unplanned Pregnancy
MICS: Multiple Indicator Cluster Surveys
RHS: Reproductive Health Surveys
UNPD: United Nations Population Definition
UNSD: United Nations Statistics Division
WHO: World Health Organization

## References

[1] Sedgh G, Bearak J, Singh S, Bankole A, Popinchalk A, Ganatra B (2016). Abortion incidence between 1990 and 2014: global, regional, and subregional levels and trends. Lancet Lond Engl.

[2] Bearak J, Popinchalk A, Alkema L, Sedgh G (2018). Global, regional, and subregional trends in unintended pregnancy and its outcomes from 1990 to 2014: estimates from a Bayesian hierarchical model. Lancet Glob Health.

[3] UN. Transforming Our World: The 2030 Agenda for Sustainable Development [Internet]. New York, NY: United Nations; 2015 [cited 2018 Nov 5]. Report No.: A/RES/70/1. Available from: http://connect.springerpub.com/lookup/doi/10.1891/9780826190123.ap02

[4] National Statistical Offices. United Nations Statistics Division; [cited 2018 Dec 10]. Available from: https://unstats.un.org/home/nso_sites/

[5] UNSD Demographic Yearbook [Internet]. United Nations Statistical Division; [cited 2018 Dec 10]. Available from: https://unstats.un.org/unsd/demographic-social/products/dyb/

[6] United Nations, Department of Economic and Social Affairs, and Population Division (2015). Estimates and Projections of the Number of Women Aged 15-49 Who Are Married or in a Union: 2015 Revision.

[7] United Nations, Department of Economic and Social Affairs, Population Division. World Contraceptive Use 2015 (POP/DB/CP/Rev2015) [Internet]. [cited 2017 Jul 6]. Available from: http://www.un.org/en/development/desa/population/publications/dataset/contraception/wcu2015.shtml

[8] Alkema L, Kantorova V, Menozzi C, Biddlecom A. National, regional, and global rates and trends in contraceptive prevalence and unmet need for family planning between 1990 and 2015: a systematic and comprehensive analysis. Lancet. 2013;381(9878):1642–52. 10.1016/S0140-6736(12)62204-1 23489750

[9] United Nations, Department of Economic and Social Affairs, Population Division. World Population Prospects: The 2015 Revision. New York: United Nations; 2015.

[10] Sedgh G, Singh S, Hussain R (2014). Intended and unintended pregnancies worldwide in 2012 and recent trends. Stud Fam Plan.

[11] Dellicour S, Aol G, Ouma P, Yan N, Bigogo G, Hamel MJ (2016). Weekly miscarriage rates in a community-based prospective cohort study in rural western Kenya. BMJ Open.

[12] Bongaarts J (2015). Modeling the fertility impact of the proximate determinants: time for a tune-up. Demogr Res.

[13] Bongaarts J, Fertility PR (1983). Biology, and behavior: an analysis of the proximate determinants.

[14] Bongaarts J (1978). A framework for analyzing the proximate determinants of Fertility. Popul Dev Rev.

[15] Rossier C, Michelot F, Bajos N, COCON Group (2007). Modeling the process leading to abortion: an application to French survey data. Stud Fam Plan.

[16] Lindberg L, Maddow-Zimet I, Desai S, Zolna M. Reporting of Abortion in Three US Surveys. 10.1007/s13524-020-00886-4 PMC7329789 32458318

[17] Singh S, Prada E, Juarez F. The Abortion Incidence Complications Method: A Quantitative Technique. In: Methodologies for Estimating Abortion Incidence and Abortion-Related Morbidity: A Review. Guttmacher Institute & International Union for the Scientific Study of Population; 2010 [cited 2018 Dec 11]. p. 63–70. Available from: https://www.guttmacher.org/sites/default/files/pdfs/pubs/compilations/IUSSP/abortion-methodologies.pdf

[18] Barret G, Smith SC, Wellings K (2004). Conceptualisation, development, and evaluation of a measure of unplanned pregnancy. J Epidemiol Community Health.

[19] Kantorová V (2013). National, regional and global estimates and projections of the number of women aged 15 to 49 who are married or in a union, 1970–2030.

[20] World Marriage Data 2017 . New York: Nations, Department of Economic and Social Affairs, Population Division; 2017 [cited 2018 Nov 5]. Available from: http://www.un.org/en/development/desa/population/publications/pdf/marriage/Metadata_World-Marriage-Data-2017.pdf

